# Nuclear Overhauser spectroscopy in hyperpolarized water – chemical *vs.* magnetic exchange†

**DOI:** 10.1039/d2cc03735a

**Published:** 2022-09-21

**Authors:** Ludovica Martina Epasto, Philipp Honegger, Kateryna Che, Fanny Kozak, Florian Jörg, Christian Schröder, Dennis Kurzbach

**Affiliations:** University of Vienna, Faculty of Chemistry, Department of Biological Chemistry Währingerstr. 38 1090 Vienna Austria dennis.kurzbach@univie.ac.at; University of Vienna, Doctoral School in Chemistry (DoSChem) Währingerstr. 42 1090 Vienna Austria; University of Vienna, Faculty of Chemistry, Department of Computational Biological Chemistry Währingerstr. 17 1090 Vienna Austria; Department of Systems Biology, Harvard Medical School 200 Longwood Avenue Boston MA 02115 USA

## Abstract

Dissolution dynamic nuclear polarization (dDNP) is a versatile hyperpolarization technique to boost signal intensities in nuclear magnetic resonance (NMR) spectroscopy. The possibility to dissolve biomolecules in a hyperpolarized aqueous buffer under mild conditions has recently widened the scope of NMR by dDNP. The water-to-target hyperpolarization transfer mechanisms remain yet unclear, not least due to an often-encountered dilemma of dDNP experiments: The strongly enhanced signal intensities are accompanied by limited structural information as data acquisition is restricted to short time series of only one-dimensional spectra or a single correlation spectrum. Tackling this challenge, we combine dDNP with molecular dynamics (MD) simulations and predictions of cross-relaxation rates to unravel the spin dynamics of magnetization flow in hyperpolarized solutions.

## Abstract 2

How to boost NMR signals of non-labile protons in hyperpolarized solutions.

## Graphical abstract

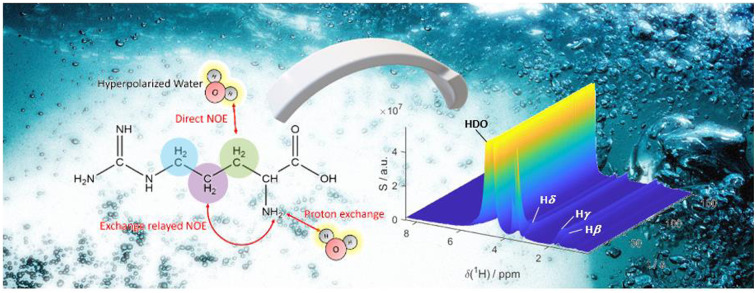

Spin hyperpolarization denotes a population distribution between nuclear spin levels far from thermal equilibrium. Such a state invokes strongly improved signal amplitudes, often over 10 000-fold enhanced, compared to conventional nuclear magnetic resonance (NMR) spectra. Various methods, from para hydrogenation to dynamic nuclear polarization (DNP) to optical pumping,^1^ enable the creation of hyperpolarized spin states for a wide array of applications. The use of hyperpolarized water to boost signal intensities in biomolecular NMR spectra has recently received ample attention^2–5^ as dissolving a target biomolecule in a hyperpolarized buffer enables new approaches at substantially enhanced sensitivity: residue- and time-resolved protein NMR,^6^ 2D and 3D correlation spectra,^7^ protein folding and folding monitoring,^8^ membrane interactions,^9^ structural dynamics,^10^ and exchange processes.^11^ However, the understanding of the hyperpolarization transfer mechanism^12^ from the buffer to the targets remained incomplete; not least as data acquisition in the, most often used, dissolution DNP (dDNP) experiments is typically limited to short periods of 1–2 min that do not allow for determining the exchange pathways *via* complex pulse sequences. Important insights into hyperpolarization transfer by nuclear Overhauser effects (NOE) between water protons and molecules dissolved therein have, *e.g.*, been achieved by Marco-Rius *et al.* (^13^C to ^1^H)^13^ or Hu *et al.* (^1^H to ^1^H or ^19^F).^14^ To this understanding, we here add a description of the interplay between direct solvent-solute NOE and exchange-relayed NOE in hyperpolarized water; an interesting phenomenon which invokes shifted weights of the different contributions to the magnetization flow as NOE and chemical exchange are differently affected by spin-hyperpolarization. Herein, we provide an understanding of these effects and, hence, also of the transient signal enhancements in biomolecular dDNP. In a dDNP experiment, the sample is *ex situ* pretreated to boost the spin resonances before transfer to an NMR spectrometer for detection. As the resulting spin-hyperpolarization after the transfer constitutes a non-equilibrium state, its lifetime is necessarily limited. We here demonstrate how to unravel the active transfer mechanisms despite a short time window by integrating dDNP experiments^1,15,16^ with cross-relaxation rates^17,18^ obtained from molecular dynamics (MD) trajectories; providing formerly inaccessible details.

Experimentally, we employed the dDNP protocol detailed in ref. 19 (see the ESI†). In brief, we hyperpolarized a water-glycerol mixture^20^ using a prototype system^21^ operating at a temperature of *T*_DNP_ = 1.4 K and a magnetic field of *B*_0,DNP_ = 6.7 T. After build-up of the ^1^H spin hyperpolarization, the sample was dissolved with a burst of D_2_O and transferred to a conventional 11.7 T NMR spectrometer for detection at 298 K. The hyperpolarized sample was then mixed in the NMR tube waiting in the spectrometer with a solution of the target molecules under study. After mixing, the hyperpolarization is readily transferred from the water to the target. As a model target to study the transfer mechanisms, we chose the ubiquitous amino acid arginine due to its widespread use and its variety of labile as well as non-exchanging protons.

Fig. 1 outlines the potential polarization transfer pathways: chemical proton exchange, direct water-to-target NOE, and exchange-relayed NOE. Phenomenologically, the magnetization transfer upon dissolution in hyperpolarized water results in a transient change of the arginine ^1^H NMR intensities. Fig. 2a and b show the resulting data. Detection started 2 s after mixing to allow the solution to settle before acquisition. The resonances of H_2_O, and Arg-H^β^, H^γ^, as well as H^δ^ can be discerned. However, the strong water resonance covered H^α^. As hyperpolarization is a non-equilibrium state, the water signal decays exponentially to its thermal equilibrium value within *ca.* 40 s (Fig. 2c). During this period, the non-equilibrium polarization is transferred to the target. We observed that for *t* < 5 s, all arginine signal intensities are reduced compared to thermal equilibrium Fig. 2b. Then, the signals rise over their equilibrium values during a time of *ca.* 40 s before reaching the thermal equilibrium. Fig. 2d and e show the negative signal enhancement computed as 1/*ε* where *ε* corresponds to the ratio of the signal amplitude *S*(*t*) at time *t* and the thermal equilibrium amplitude *S*_TE_. All observed arginine protons, Arg-H^β^, H^γ^, and H^δ^ do not chemically exchange with the solvent and yet show substantial signal amplitude changes during this period. Note that the water signal decay is strictly monotone, which outrules biases in signal intensities due to incomplete mixing or convection. The ESI,† contain similar data for poly-aspartate, as a proof-of-concept that the reported method can be applied to other amino acids and macromolecular systems, too.

**Fig. 1:**
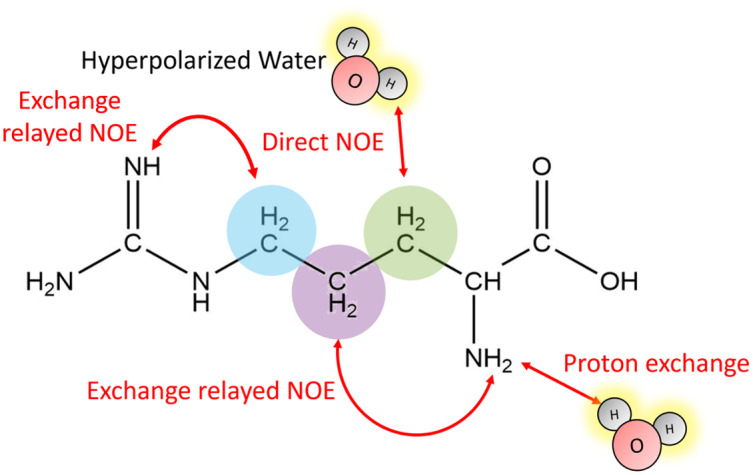
Hyperpolarization transfer pathways between water and arginine: direct nuclear NOE between the solvent and the target, chemical proton exchange of labile moieties, or exchange-relayed NOE. The β-,γ and δ-hydrogens are indicated by the green, violet and blue spheres, respectively.

**Fig. 2:**
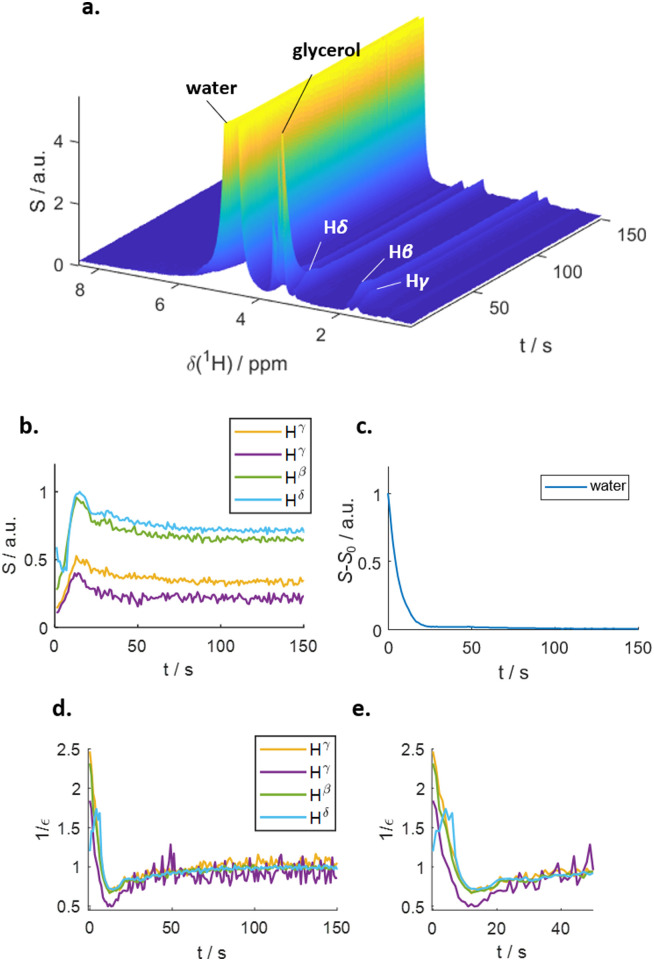
(a) Time series of proton NMR spectra of arginine in hyperpolarized water. *t* = 0 corresponds to the start of the acquisition period. Next to the aliphatic protons, the water and glycerol lines are visible. (b) Intensities of the aliphatic arginine protons. An initial negative enhancement for 5 s can be discerned, prior to a positive enhancement at *t* > 5 s. (c) Intensity of the water signal. The decay is strictly monoexponential. (d) Negative signal enhancements 1/*ε vs.* time. (e) The first 50 s of panel d.

The observed behavior correlates with the well-documented^22,23^ superposition of a negative direct NOE between the solvent and the target that dominates at the beginning of the time trace and reduces the arginine signal amplitudes and the effects of chemical exchange and exchange-relayed NOE that dominates during the later stages of the trajectory and increases the signal amplitudes. The initial negative enhancements *ε*_*i*_ are listed in Table 1.

**Table 1:** Experimental initial signal enhancements 1/*ε*_*i*_ and computational values for the arginine hydrogens. For the coordination number and the residence time *τ* only water molecules closer than six Å to an arginine hydrogen were considered

Atom	1/*ε*_*i*_ [a.u.]	*σ*_L_ [a.u.]	Distance < 6 Å	
			# H_2_O	*τ* [ps]
H^α^	—	4.98	24	8.3
H^β^	2.30	4.95	24	7.6
H^γ^	2.22	4.70	23	7.2
H^δ^	1.24	4.55	23	6.5

The differences in build-up and decay rates in Fig. 2b–e result from proton position-dependent efficiencies of hyperpolarization transfer, either *via* direct or exchange-relayed NOE. To corroborate this interpretation as well as that of the negative signal enhancement at *t* < 5 s as direct NOE between solvent and target, we employed MD simulations (see ESI† for details) to calculate the direct water-arginine polarization exchange using Redfield relaxation theory.^24^

The first 20 ns of each trajectory were discarded to avoid the inclusion of possible slow equilibration artifacts. To compute cross-relaxation rates, five hundred starting points were evenly spaced along the trajectory for each correlation function. At each starting point, the distance of the spin pairs was minimized by centering the reference amino acid spin in the simulation box. Subsequently, the coordinates of all molecules were unfolded to undo the coordinate jumps caused by periodic boundary conditions, restoring the natural diffusive motion of the spin pairs. The resulting correlation functions were Fourier transformed using NumPy, and the cross-relaxation rates were averaged over all arginines.

For the computation of the NOE, we capitalized on the fact that the magnetization transfer between two interacting spins I and S with a nuclear spin of 1/2 (*e.g.* an arginine hydrogen atom and a water hydrogen atom) takes place strictly *via* a dipole-coupled mechanism. The corresponding time correlation function1
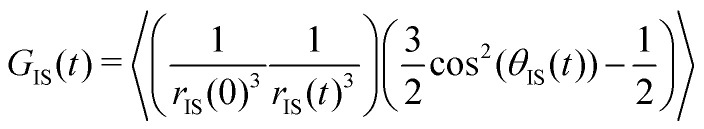
depends on two terms: The first is a function of the vector *r⃑*_IS_ joining the two interacting spins I and S; its randomization rate is determined by the translational diffusion of these two spins towards or away from each other. Typically, this is the numerically dominant term in intermolecular NOEs.^25^ The second term depends on the angle *θ*_IS_ swept by the spin-joining vector during time *t*. This term represents the gyration of the two spins around a common center. The spectral density *J*_IS_(*ν*) can be obtained by real-part Fourier transformation2
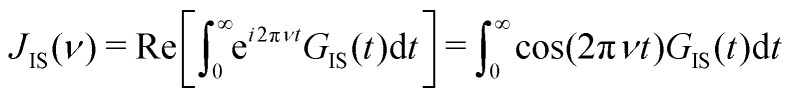
and yields the laboratory-frame cross relaxation rate *σ*^NOESY^_L_*via*^26,27^

3 *σ*^NOESY^_L_(*ν*) = 0.6*J*_IS_(*ν*_I_ + *ν*_S_) − 0.1*J*_IS_(|*ν*_I_ − *ν*_S_|)

as a function of the Larmor frequencies *ν*_I_ and *ν*_S_ of the interacting nuclei I and S. Choosing the field used in our experiments for detection (11.7 T corresponding to a Larmor frequency of 500 MHz for protons; see ESI,† Fig. S2), we could extract the relative efficiencies of the solvent NOE for Arg-H^α^, H^β^, H^γ^, as well as H^δ^.

The spectral density function in eqn (2) is a function of the distance of the interacting species. The distance therefore also affects *σ*^NOESY^_L_.^28^ The most important contributions to *σ*^NOESY^_L_ should be covered within a distance of 6 Å.^28^ Using this limit, we counted approximately 24 water molecules in the vicinity of the H^α^, H^β^, H^γ^ and H^δ^ protons. The corresponding residence times (see Table 1) correlate well with the NOE relaxation rate as shown in the radio plot in Fig. 3.

**Fig. 3:**
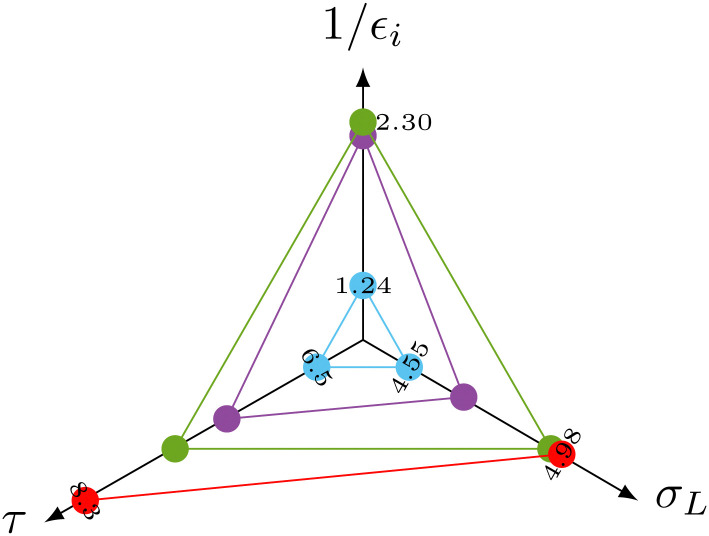
Radio plot of the correlation between the experimental negative signal enhancement immediately after start of the detection 1/*ε*_*i*_, the NOE relaxation rate *σ*_L_ and the residence time *τ* of the interaction water molecules. H^α^ (red) was masked in the experiments by the broadened water line.

More importantly, the measured initial negative signal enhancements also correlate with the computed values (Fig. 3) corroborating our interpretation of the signal reduction in the dDNP experiments as direct solvent-to-target NOE. The H^β^ (green dots) atoms showing the strongest initial negative enhancement 1/*ε*_*i*_ lead to the highest rates *σ*_L_. In contrast, water molecules near the arginine H^δ^ (blue dots) led to a much reduced 1/*ε*_*i*_ as accompanied by slower NOE transfer and shorter residence times. H^γ^ (purple dots) consistently laid between H^β^ and H^δ^ for all three parameters.

However, the correlation of computed and experimental data suffers from the drawback that chemical exchange is neglected in our simulations. Hence, we further complemented our conclusions with conventional NOESY (Nuclear Overhauser Enhancement SpectroscopY). We found that both, direct NOE between the water and the different arginine protons as well as exchange processes, are effectively transferring nuclear spin polarization (Fig. 4; see the ESI† for other mixing times). For all mixing times, negative cross-peaks (relative to negative diagonal peaks) between the labile H^N^ and H^η^ resonances of arginine and the water indicate chemical proton exchange. In addition, we observed cross-peaks between the water and H^β^, H^γ^, as well as H^δ^ indicating direct NOE between arginine and solvent. Intramolecular NOE between all involved arginine protons could be observed, as expected (Fig. 4).

**Fig. 4:**
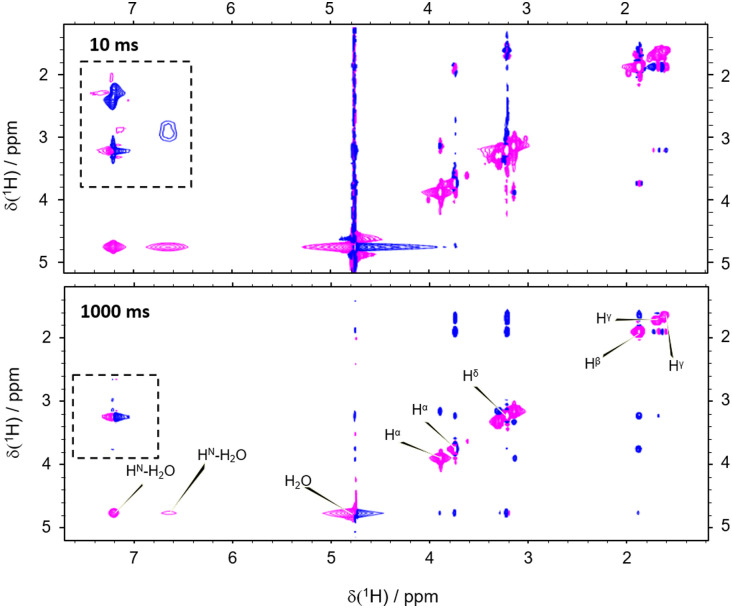
Thermal equilibrium NOESY with a mixing time of 10 ms (top) and 1 s (bottom). Positive cross-peaks between the water and the non-exchangeable protons evidence intermolecular NOE. Cross-peaks between the water and the labile protons indicate proton exchange. The dashed box highlights magnetization transfer between exchanging and aliphatic protons, *i.e.*, exchange-relayed NOE.

Importantly, the NOESY show the simultaneous action of chemical proton exchange and intramolecular NOE leading to exchange-relayed NOE. This is crucial for understanding the hyperpolarization transfer. Since the solvent NOE features an opposed sign relative to the diagonal peaks, an exchange-relayed NOE is necessarily responsible for the positive signal enhancements of the non-exchangeable protons at *t* > 5 s. The observation of cross-peaks between the signals of the labile H^N^ protons and H^β^, H^γ^, as well as H^δ^ confirms the flow of magnetization from exchanging to non-exchanging moieties (Fig. 4 dashed rectangle).

Summarizing, all three pathways (direct solvent NOE, chemical exchange, as well as exchange relayed NOE) depicted in Fig. 1 can transfer hyperpolarization from the water to a target molecule to boost NMR spectra of biomolecules in a hyperpolarized buffer. For arginine at *B*_0_ = 11.7 T, we find negative signal enhancements for all non-labile protons by direct NOE for *t* < 5 s after dissolution in hyperpolarized water and positive enhancements for *t* > 5 s by exchange-relayed NOE. As water is a ubiquitous solvent, the reported mechanisms can be readily extended and exploited for other target molecules. The combination of dDNP and computational approaches can thereby be a powerful asset to enlighten both, structural as well as spin dynamics of hyperpolarized solutions. It should be noted that in experiments that detect vividly exchanging protons, as typical for ^1^H–^15^N cross-peaks or ^15^N-edited ^1^H signals of peptides, nucleic acids, and proteins, the contribution of the negative, direct solvent NOE is canceled by the much more effective proton exchange pathway reaching enhancements of *ε* > 100. However, for non-labile protons, this effect cannot be neglected and competes with the exchange-relayed pathway. The importance of exchange-relayed effects is further supported by earlier work^6^ that indicated that backbone ^1^H–^15^N signal enhancements are strongest for residues with protic side chains. This observation can readily be explained with exchange-relayed NOE between the side chain and backbone protons.

All raw data are available under https://doi.org/10.5281/zenodo.7113098.

LME, KC and FK performed the experiments. PH developed the software to analyze the MD trajectories. LME, PH and FJ performed simulations. LME, CS and DK analyzed data and conceived the study.

The authors acknowledge support from the NMR core facility of the Faculty of Chemistry, University Vienna, an ERC StG (No 801936), and 2 FWF grants (no. P-33338 N, J-4537 and I4383N).

## Conflicts of interest

There are no conflicts to declare.

## Supplementary Material

CC-058-D2CC03735A-s001
