# Supplementary material for: Nuclear Overhauser spectroscopy in hyperpolarized water – chemical vs. magnetic exchange
Source: Chem Commun (Camb). 2022 Sep 21;58(83):11661–4. doi: 10.1039/d2cc03735a (PMC9578288; doi:10.1039/d2cc03735a)
Supplement: CC-058-D2CC03735A-s001 [file CC-058-D2CC03735A-s001.pdf]

Electronic Supplementary Material (ESI) for ChemComm.
This journal is © The Royal Society of Chemistry 2022

Nuclear Overhauser spectroscopy in hyperpolarized water - chemical vs. magnetic exchange†

Supplementary Information

Ludovica Martina Epasto,[a,b] Philipp Honegger,[c,d] Kateryna Che,[a] Fanny Kozak,[a,b] Florian Jörg,[b,c], Christian Schröder[c] and Dennis Kurzbach[a,*]

Contents

1 Experimental materials and methods **2**
1.1 DNP 2
1.2 NMR 2

2 Computational analysis of the NOE **4**

3 Additional experimental data **6**

1 Experimental materials and methods

1.1 DNP

Spin hyperpolarization of $150\,\mu$L of a flash-frozen (vitrified) $15\,$mM 4-Hydroxy-2,2,6,6-tetramethylpiperidinyloxyl (TEMPOL) solution containing 10% H_2O, 40% D_2O, and 50% glycerol-d8 was performed at $1.4\,$K and a magnetic field of $6.7\,$T in a cryostat-magnet apparatus, purchased from Cryogenic Ltd. The sample was irradiated with microwaves at a frequency of $188.08\,$GHz using a Virginia Diodes Inc. (VDI) microwave source and frequency amplifier. The hyperpolarization build-up was monitored through a $400\,$MHz Bruker Avance III spectrometer modified to operate at $285.3\,$MHz. A homemade tune and matching system was combined with the $400\,$MHz console. 1° detection pulses every $5\,$s were applied to monitor the proton polarization build-up. For DNP and sample dissolution, the system described in Ref. 1 was used.

After polarizing for $3\,$h, $5\,$mL of preheated D_2O were injected at a pressure of $1.5\,$MPa onto the sample for dissolution. The solvent was pressurized in a homemade pressure heater combined with an Arduino system, controlled by a home-written MATLAB code. The hyperpolarized solution was then transferred with pressurized helium gas at $0.6\,$MPa to an NMR tube placed into a $500\,$MHz Bruker NEO spectrometer, equipped with a Prodigy BBFO cryo-probe. In the NMR tube, the DNP sample mixed rapidly with $200\,\mu$L of 2-(N-morpholino)ethanesulfonic acid (MES) buffer ($25\,$mM NaCl, $25\,$mM MES, $100\,$mM arginine hydro chloride (ArgHCl), pH 5.5), reconstituted in D_2O waiting inside the NMR spectrometer. Therefore, the MES buffer was diluted up to a volume of $5\,$mL, with a final ArgHCl concentration of $4\,$mM. The detection was carried out employing using 1° hard pulses every second using the 'zg2d' pulse sequence implemented in Bruker TopSpin 4. The dDNP data were baseline corrected and fitted to a Lorentzian function using the MATLAB R2019a software to extrapolate signal intensities.

1.2 NMR

The series of conventional ^{1}H-^{1}H nuclear Overhauser enhanced spectroscopy (NOESY) spectra was acquired on a $600\,$MHz Bruker NEO spectrometer equipped with a cryogenically cooled TCI probe. The 'noesyfpgpphrs190' pulse sequence was used as implemented in the Bruker TopSpin 4 pulse program library. Twelve NOESY experiments with different mixing times (10, 20, 30, 40, 50, 70, 100, 150, 200, 300, 500, and $1000\,$ms) were collected on a $100\,$mM arginine solution in $600\,\mu$L MES buffer (pH 5.5.) containing 10% D_2O as field lock solvent. quadrature detection was achieved using the States-Time Proportional Phase Incrementation (TPPI) method. All NOESY spectra were measured with spectral widths of $9615.3\,$Hz in the F2 dimension and $7202.1\,$Hz in the F1 dimension. We recorded 32 scans. The processing and analysis of the collected data were carried out on Topspin 4.0.7, SPARKY, and MATLAB R2019a. The NOESY spectra were zero-filled and Fourier transformed prior to peak assignment. The arginine peaks were assigned according to SDBS entry 1143.

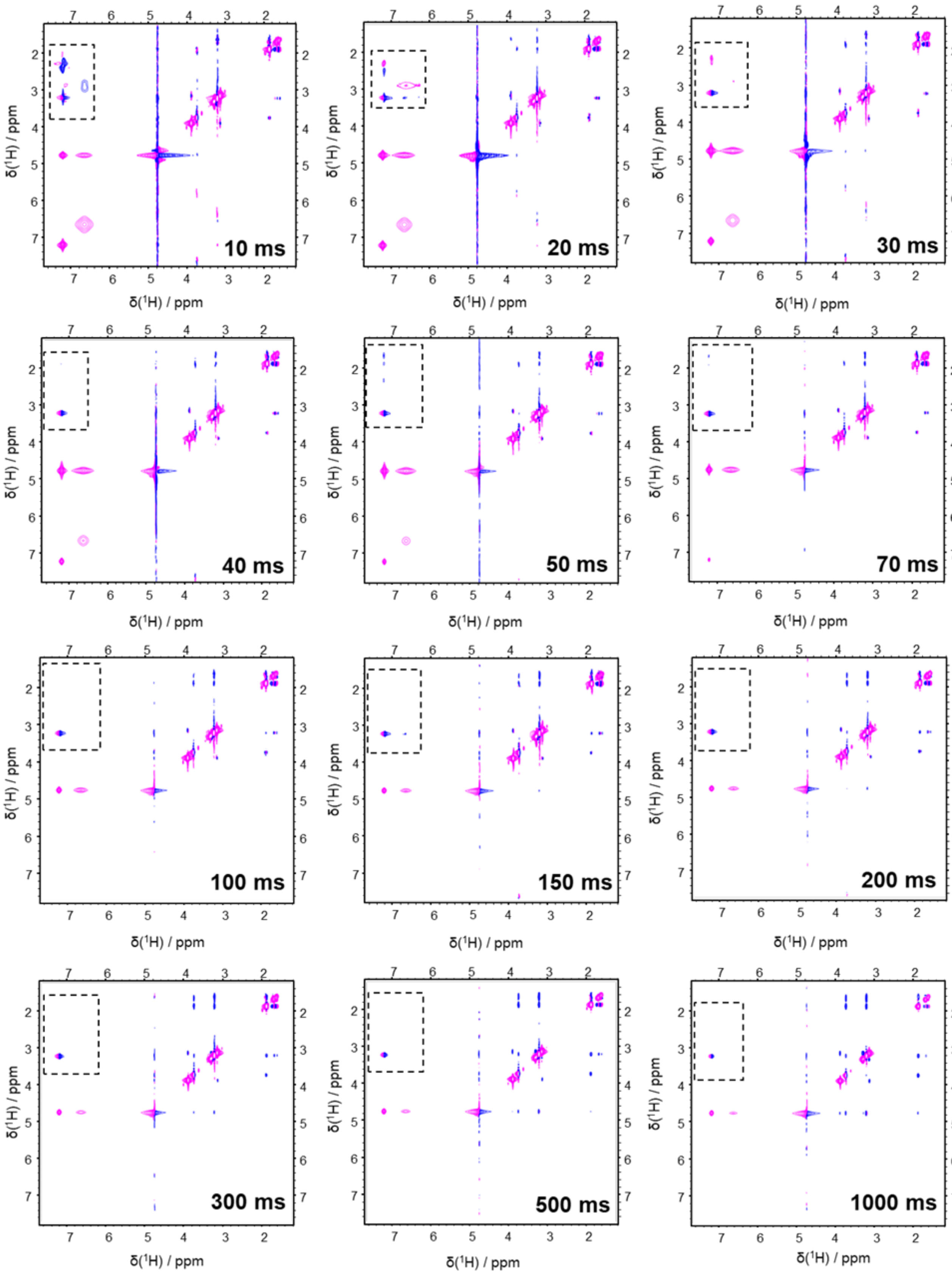

Figure S1: Thermal equilibrium NOESY experiment on a 100 mM ArgHCl solution with different mixing times (indicated on the bottom right of each spectrum).

2 Computational analysis of the NOE

We used the CHARMM36 (Chemistry at Harvard Macromolecular Mechanics 36) force field,[2–5] as implemented in CHARMM-GUI[6] for MD simulations of ten arginine and ten chloride counter ions were surrounded by 6788 water molecules (TIP3P model[7,8]). The initial intermolecular geometries were optimized by energy minimization, before equilibration for $5\,\mathrm{ns}$ as isothermal-isobaric ensembles (NpT; T=300 K, p=1 atm) until the simulation box lengths converged at a =60 Å. The trajectories were produced as isothermal-isochoric ensembles (NVT, T=300 K) using a Nosé-Hoover thermostat[9,10] with periodic boundary conditions in OpenMM.[11] High-frequency vibrations of covalent bonds involving hydrogen atoms were constrained using the Shake algorithm.[12] Non-bonded interactions were cut off at 12 Å, and electrostatic interactions were treated using the Ewald method. The simulation trajectories were read into a Python3-based analysis program using the MDAnalysis module.[13,14] Computational bottlenecks were accelerated using the high-performance hybrid language Cython.

The NOE is a magnetization transfer phenomenon mediated by the nuclear dipoles and is a function of the distance and not the chemical connectivity of the interacting nuclei I and S. Using Redfield theory[15] and Solomon's differential equations[16]

$$\frac{d\langle \Delta I_z\rangle}{dt} = \rho_{II}\langle \Delta I_z\rangle + \sigma_{SI}\langle \Delta I_z\rangle \tag{1}$$

$$\frac{d\langle \Delta S_z\rangle}{dt} = \sigma_{IS}\langle \Delta S_z\rangle + \rho_{SS}\langle \Delta S_z\rangle \tag{2}$$

the kinetics of the two spins I and S can be related to the self- (ρ) and cross-(σ) relaxation rates. Following Redfield theory, these rates can be obtained from the spectral density function $J(\nu)$:

$$\rho_{II} = 0.6J(\nu_I+\nu_S) + 0.3J(\nu_S) + 0.1J(\nu_I-\nu_S) \tag{3}$$

$$\sigma_{IS}^{NOE} = 0.6J(\nu_I+\nu_S) - 0.1J(\nu_I-\nu_S) \tag{4}$$

$$\sigma_{IS}^{ROE} = 0.3J(\nu_S) + 0.2J(\nu_I-\nu_S) \tag{5}$$

with $\rho_{II} = \sigma_{IS}^{NOE} + \sigma_{IS}^{ROE}$. As shown in Ref.[17], the ratio $\sigma_{IS}^{NOE}/\sigma_{IS}^{ROE}$ is not plagued by long-range contributions whereas the individual σ_{IS}^{NOE} and σ_{IS}^{ROE} are affected.

A similar effect is also visible in our water residence times. In Table S1, the residence times of water molecules within four Å and six Å of the respective arginine hydrogen are displayed.

Table S1: Experimental initial signal enhancements $1/\epsilon_i$ and computational values for the arginine hydrogens. For the coordination number and the residence time τ only water molecules with hydrogens closer than four/six Å to the corresponding arginine hydrogen were taken into account in the simulation.

Atom	$1/\epsilon_i$	σ_L	σ_L/σ_R	distance < 4 Å		distance < 6 Å	
	[a. u.]	[a. u.]		# H_2O	τ[ps]	# H_2O	τ[ps]
H^α	—	4.98	0.986	4.6	4.0	24	8.3
H^β	2.30	4.95	0.984	3.7	1.8	24	7.6
H^γ	2.22	4.70	0.981	3.9	1.9	23	7.2
H^δ	1.24	4.55	0.981	4.2	2.9	23	6.5

Using the larger distance criterion r <6 Å, the cross-relaxation rate $\sigma^{NOE} = \sigma_L$ shows the same trend as the residence times: The longer σ_L, the slower τ. Increasing the distance criterion results in a larger overlap of the water neighborhood of the argenine hydrogens and consequently blurs the trend.

Interestingly, the ranking of the residence times is reversed for the shorter distance criterion of four Å (see Table S1). Here, only more or less direct water neighbors of the argenine hydrogens are taken into account. This situation resembles the short-range effect of the ratio $\sigma_L^{NOESY}/\sigma_R^{ROESY}$ which has been used for protein surface hydration mapping.[18] However, since arginine is a small molecule and all the water residence times are all similarly short, the ratio $\sigma_L^{NOESY}/\sigma_R^{ROESY}$ becomes almost uniform as has been previously found for disaccharide hydration.[19]

It should be noted that the detected samples contained 1.4% glycerol-d8, which was used as (necessary) glassing agent in the dDNP experiments. It is well-documented that glycerol can influence the hydration shell of biomolecules. However, concentrations of >10% v/v are typically required to observe such effects.[20] Hence, in our MD simulations, the presence of glycerol was neglected. The correspondence between the computed and

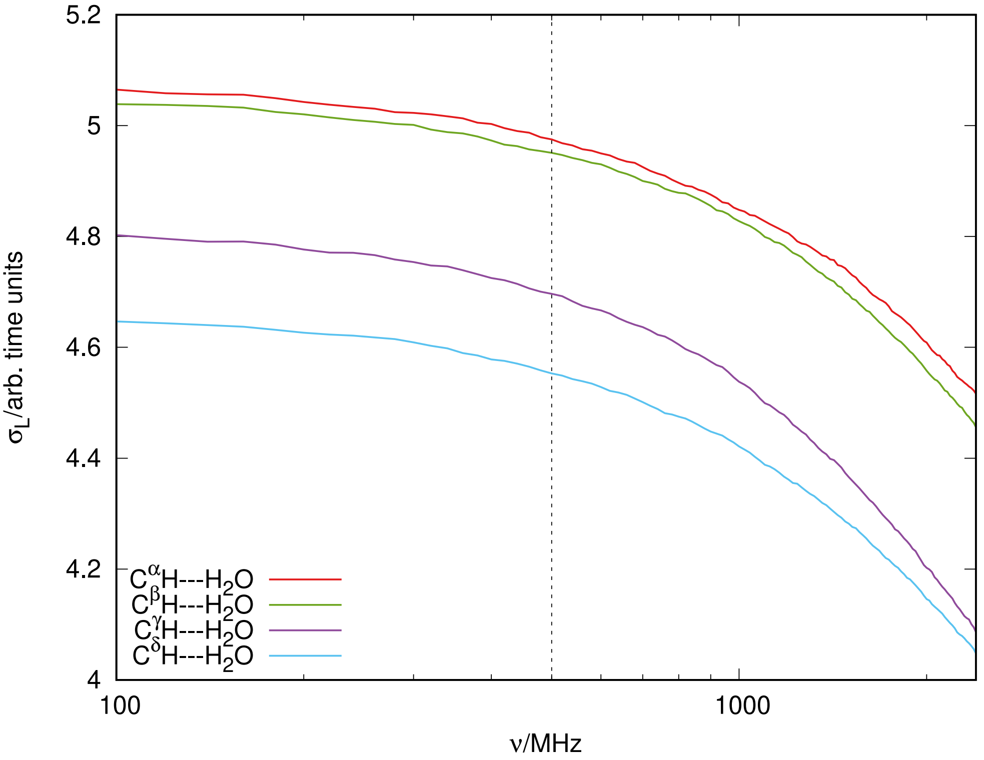

Figure S2: Predicted solvent NOE rates (colored solid lines) at different resonance frequencies for each non-exchangeable arginine proton. The black dashed line indicates the experimental Larmor frequency.

experimentally observed relaxation rates justifies this simplification. Nevertheless, this potential bias of possible changes in hydration shells should be considered when precise quantifying computed NOE rates is required. Likewise, the presence of D_2O in the hyperpolarized samples was not considered during the MD simulations. However, deuteron exchange and dilution of water by D_2O can indeed impact NOE rates as well as proton exchange efficiencies. This potential bias could experimentally yet not be confirmed as only hyperpolarized protons were detected. However, again, it should be kept in mind that the degree of deuteration could potentially bias the MD simulations when aiming to extract precise NOE rates.

3 Additional experimental data

To showcase the application of our method to other biological systems and verify that the approach is suitable for different amino acids, too, we performed similar dDNP experiments to study the transfer of polarization from water to polyaspartate (pAsp) at a concentration of 1 mg / mL. We followed the same protocol outlined above. We observed that, while the signal of the H^{β} protons is readily identified at 2.6 ppm, the signal of the H^{α} proton was again covered by the broad water peak. The enhanced signals of the amide protons were detected at 8.2 ppm. The hyperpolarized proton signals decay within approximately 20 seconds. As for the arginine dDNP experiments, $t = 0$ corresponds to the start of acquisition. The amide proton signal decays monotonic, as expected since the enhancement is based primarily on chemical proton exchange. Contrarily, the H^{β} signal reaches its maximum intensity two seconds after the beginning of the detection period as a result of the slower exchange-relayed NOE rate. Importantly, the observed signal build-ups and decays differ from those observed for arginine. As the hydration shells of a single amino acid and a biomacromolecule necessarily differ, such an observation can be expected. Therefore, the possibility arises to determine intramolecular interactions or conformational changes from different solvent interaction.

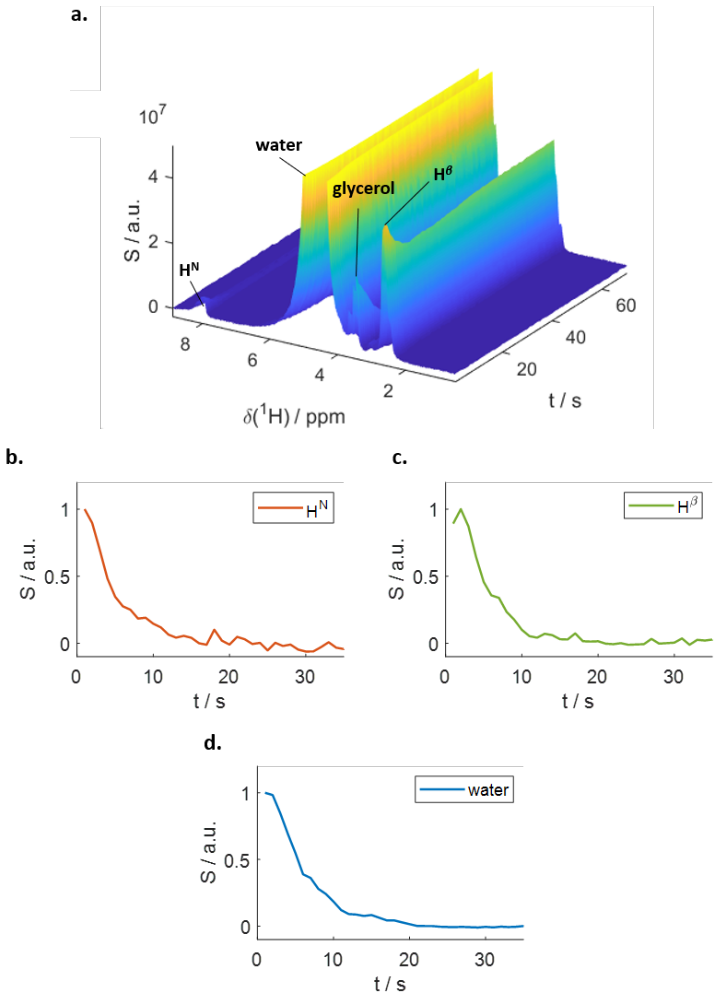

Figure S3: Hyperpolarized water experiments with polyaspartate. a) Time series of proton NMR spectra of pAsp in hyperpolarized water. At t=0, signals of the amide protons, the water, the glycerol and the aliphatic protons are discernible. b) Intensities of the pAsp amide protons. The signal decay is strictly monoexponential . c) Intensities of the H^{β} signal. The maximum intensity of the signal is reached two seconds after start of the detection, indicating the contribution of NOE to the pAsp system. d) Intensity of the water signal.

References

[1] T. Kress, K. Che, L. M. Epasto, F. Kozak, M. Negroni, G. L. Olsen, A. Selimovic and D. Kurzbach, *Magn. Reson.*, 2021, **2**, 387–394.

[2] D. Beglov and B. Roux, *J. Chem. Phys.*, 1994, **100**, 9050.

[3] A. D. MacKerell Jr., D. Bashford, M. Bellott, R. L. Dunbrack Jr., J. D. Evanseck, M. J. Field, S. Fischer, J. Gao, H. Guo, S. Ha, D. Joseph-McCarthy, L. Kuchnir, K. Kuczera, F. T. K. Lau, C. Mattos, S. Michnick, T. Ngo, D. T. Nguyen, B. Prodhom, W. E. Reiher, B. Roux, M. Schienkrich, J. C. Smith, R. Stote, J. Straub, M. Watanabe, J. Wlorklewicz-Kuczera, D. Yin and M. Karplus, *J. Phys. Chem. B*, 1998, **102(18)**, 3586–3616.

[4] A. D. MacKerell Jr., M. Feig and C. L. Brooks III., *J. Am. Chem. Soc.*, 2004, **126**, 698–699.

[5] R. B. Best, X. Zhu, J. Shim, P. E. Lopes, J. Mittal, M. Feig and A. D. MacKerell Jr., *J. Chem. Theory Comput.*, 2012, **8(9)**, 3257–3273.

[6] S. Jo, T. Kim, V. G. Iyer and W. Im, *J. Comput. Chem.*, 2008, **29**, 1859–1865.

[7] W. L. Jorgensen, J. Chandrasekhar, J. D. Madura, R. W. Impey and M. L. Klein, *J. Chem. Phys.*, 1983, **79**, 926–935.

[8] E. Neria, S. Fischer and M. Karplus, *J. Chem. Phys.*, 1996, **105**, 1902–1921.

[9] S. Nose, *J. Chem. Phys.*, 1984, **81**, 511.

[10] W. G. Hoover, *Phys. Rev.*, 1985, **A31**, 1695.

[11] P. Eastman, J. Swails, J. D. Chodera, R. T. McGibbon, Y. Zhao, K. A. Beauchamp, L.-P. Wang, A. C. Simmonett, M. P. Harrigan, C. D. Stern, R. P. Wiewiora, B. R. Brooks and V. S. Pande, *PLoS Comput. Biol.*, 2017, **13**, e1005659.

[12] J.-P. Ryckaert, G. Ciccotti and H. J. C. Berendsen, *J. Comput. Phys.*, 1977, **23**, 327.

[13] N. Michaud-Agrawal, E. J. Denning, T. B. Woolf and O. Beckstein, *J. Comput. Chem.*, 2011, **32**, 2319–2327.

[14] R. J. Gowers, M. Linke, J. Barnoud, T. J. E. Reddy, M. N. Melo, S. L. Seyler, D. L. Dotson, J. Domanski, S. Buchoux, I. M. Kenney and O. Beckstein, *Proc. 15th Python Sci. Conf.*, 2016, 102–109.

[15] A. G. Redfield, *IBM J. Res. Dev.*, 1957, **1**, 19–31.

[16] I. Solomon, *Phys. Rev.*, 1955, **99**, 559.

[17] P. Honegger and O. Steinhauser, *Phys. Chem. Chem. Phys.*, 2019, **21**, 14571–14582.

[18] N. V. Nucci, M. S. Pometun and A. J. Wand, *Nature Structural & Molecular Biology*, 2011, **18**, 245–249.

[19] E. Heid, P. Honegger, D. Braun, A. Szabadi, T. Stankovic, O. Steinhauser and C. Schröder, *J. Chem. Phys.*, 2019, **150**, 175102.

[20] M. Hirai, S. Ajito, M. Sugiyama, H. Iwase, S.-i. Takata, N. Shimizu, N. Igarashi, A. Martel and L. Procar, *Biophys. J.*, 2018, **17**, 313–327.
